# COVID-19’s shadow on families: A structural equation model of parental stress, family relationships, and child wellbeing

**DOI:** 10.1371/journal.pone.0292292

**Published:** 2023-10-12

**Authors:** Antje von Suchodoletz, Jocelyn Bélanger, Christopher Bryan, Rahma Ali, Sheikha R. Al Nuaimi

**Affiliations:** 1 Department of Psychology, New York University Abu Dhabi, Abu Dhabi, United Arab Emirates; 2 New York University Global TIES for Children Research Center, Abu Dhabi, United Arab Emirates; Aalborg University, DENMARK

## Abstract

The present study seeks to contribute to developmental science in emergencies by investigating associations between COVID-19 pandemic-related stressors, parents’ stress, family relationships, and child wellbeing. In doing so, we build on recent research that generalizes the assumptions of the Family Stress Model beyond direct economic stressors of households to macro-contextual stressors that operate at the societal level. In the case of our study, these stressors relate to the COVID-19 pandemic, such as health risks and confinement-related stresses. Participants were 783 parents of young children (75% female, *M*_age_ = 34.61 years) residing in the United Arab Emirates. They completed an online survey in Fall 2020 and Spring 2021, measuring how the pandemic impacted their lives and the lives of their child (*M*_age_ = 47.54 months). A subsample of parents (*n* = 96) completed the survey for two children. Structural equation modeling showed that pandemic-related stressors contributed to higher stress among parents which, in turn, resulted in lower parent-reported child wellbeing at various times during the pandemic. Family relationships mediated the association between parents’ stress and child wellbeing. The present study contributes to our understanding on how large-scale disruption due to COVID-19 pandemic-related stressors gets inside the family, the strength and direction of associations (concurrently and over time), and the timing of mechanisms that impact family processes. The results highlight the need to support families with young children in managing disruptions due to emergencies, such as a global public health crisis, and to determine ways of preventing longstanding consequences on family structures and children’s lives.

## Introduction

Governments around the world implemented a range of measures, including movement restrictions, social distancing, and partial or full closure of schools and businesses, in response to the COVID-19 outbreak. Although these measures helped decrease transmission intensity, they imposed large-scale social, economic, and personal costs that were particularly devastating for families and children [1–5]. Families faced sudden changes to childcare and schooling arrangements along with work-from-home requirements. Balancing care and job commitments was even more challenging due to the disruption of social support systems [6]. In addition, families experienced income losses, pushing households–even those in high-income countries–into financial crisis [7]. Together with the threats to physical health from the COVID-19 virus itself, the financial insecurity, caregiving burden, and confinement-related stresses hindered the functioning of family systems, resulting in consequences that may be longstanding, both on parents’ and children’s lives [6].

Research quickly responded to the public health crisis by describing the impact of the pandemic on family functioning and child wellbeing across the world [2, 8–13]. What has yet to be understood is how COVID-19 disruptions get inside the family [5, 14]. Such knowledge is important for planning and implementing policies and interventions aimed at supporting families during periods of public health and other crises. Initial longitudinal studies found that associations between parental strain due to COVID-19 pandemic-related stresses and child wellbeing unfold and change over time [5]. Such temporal dynamics highlight the close link of child and parent psychological functioning [5, 15]. They also underscore that macro-contextual stressors that operate at the societal level (such as COVID-19 related protocols and risks) impact micro-contextual processes at the family level [5]. Consistent with developmental systems theorizing, our study was guided by the Family Stress Model (FSM), which argues that economic hardships and pressures can influence child outcomes through their impact on parental stress, marital relationship, and parenting behavior [16]. While the FSM is primarily focused on economic stressors, Masarik and Conger [16] recognize that acute and chronic stressors may also increase the risk for both parents and children to encounter relational problems. This suggests that the FSM can be a useful lens to identify mechanisms through which pandemic stress may impact family processes and children’s development [6, 14, 17].

**Fig 1:**
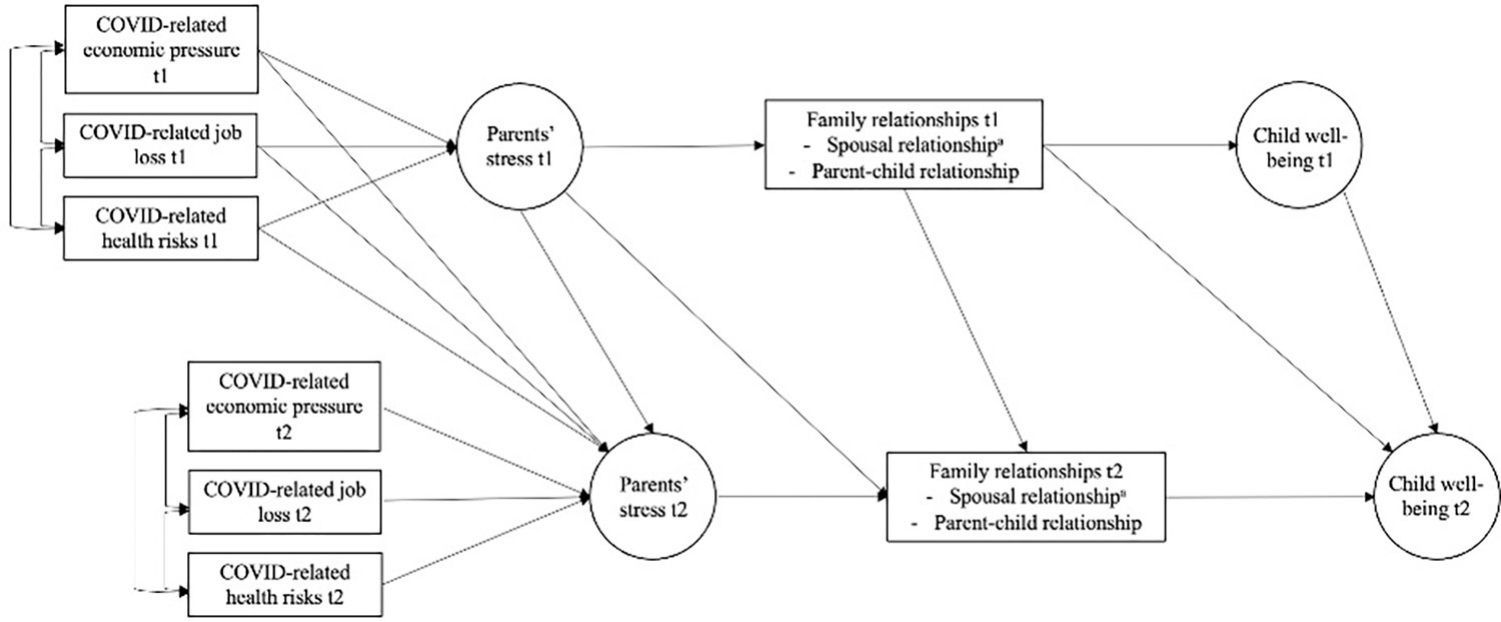
Conceptual model for associations between COVID-19-related hardships and pressures, parents’ stress, family relationships, and child wellbeing.

The present study seeks to contribute to developmental science in emergencies by testing associations between COVID-19 pandemic-related stressors, parents’ stress, family relationships, and child wellbeing at the family level (Fig 1). In doing so, we build on recent research that generalizes the assumptions of the FSM beyond direct economic stressors of households to macro-contextual stressors that operate at the societal level, such as the COVID-19 pandemic [14], and on frameworks of family wellbeing [6] and life course perspectives [17]. Our findings contribute to evidence on the mechanisms underlying the impact of the pandemic on family and child wellbeing.

### Theoretical considerations

The FSM emphasizes that child adjustment problems are exacerbated when economic hardships are placed on family processes. Economic hardships can be manifold and defined objectively as well as subjectively [18]. Objective measures are factual reports of life circumstances (such as employment, and income). Subjective measures, in contrast, describe one’s perception of life circumstances and economic situation [18, 19]. Together, hardships (such as job losses, reduced income, income instability, and unstable work conditions) can result in unstable conditions for families with impacts on family functioning [16]. A recent meta-analysis found that both objective and subjective economic hardships showed associations with subjective wellbeing. Importantly, the links were stronger for subjective measures [18]. In the context of the COVID-19 pandemic, many families experienced economic hardships, particularly job loss and financial insecurity [6, 7, 20]. For example, a study from India found that these two aspects of economic hardship were associated with poorer mental health outcomes [20]. Reflecting both the assumptions of the FSM and the pandemic-specific sources of disruptions, we used COVID-19-related job loss as objective indicator and perceived economic pressure (i.e., perception of potential economic consequences for one’s family) as subjective indicator in our conceptual model (Fig 1). Addressing the specifics of the global health crisis, perceived pandemic-related health risks were included as additional indicator of hardship.

In accordance with the FSM, we assumed that objective and subjective hardships experienced by families during the pandemic drained parents’ mental and emotional resources and contributed to higher parental stress (Fig 1). Research shows that parents’ risk of experiencing stress increased substantially after the COVID-19 outbreak began in early 2020 [12, 21–23]. It has been reported that parents feel “far less energetic, more stressed, tired, angry, listless, nervous, bored, and quite worried” [23] (p. 165). Common stressors were changes in daily routines, anxiety around COVID-19 and health risks, and demands related to at-home care of children [21]. Importantly, since the start of the pandemic, parents were more likely to feel stressed than adults without children [24, 25]. According to Sprang and Silman [26], pandemic disasters create conditions that families and children find traumatic. Reports from 398 parents with varying disease-containment experiences following the H1N1 outbreak in 2009 indicated that 25% of parents met criteria for PTSD. In addition, significant associations between PTSD symptoms in parents and children were reported [26], suggesting that pandemic stressors disrupt family wellbeing [6].

The FSM further assumes that economically-influenced parents’ stress can cause problems in the partner relationship by increasing partner conflict and negative perceptions of the relationship and decreasing satisfaction and perceived spousal support [16]. Similar processes have been used to explain relationship stress in the context of COVID-19 [6, 27]. Previous literature suggests that stressors associated with disasters interfere with adaptive dyadic relationship processes. The unknown duration of the current pandemic and the pervasive uncertainty and fear regarding its impacts may destabilize relationships [27]. A growing body of evidence supports the conclusion that parents’ stress during the COVID-19 pandemic is adversely affecting spousal relationships (e.g., Singapore [28]; Israel [29]; Iran [30]; US/Canada [31]).

In the context of the FSM, parents’ stress and spousal relationship problems are assumed to increase disrupted parenting, leading to insensitive and unresponsive parenting behaviors, which is thought to result in lower quality of parent-child relationships [16]. The conceptual framework of family wellbeing builds on these assumptions and argues that heightened parents’ stress due to the COVID-19 pandemic changes family relationships due to the interconnections among family members [5]. Similarly, in life course perspectives, the construct of linked lives is used to argue that the COVID-19 pandemic is not experienced in a vacuum, but children navigate it together with important others, in particular their parents, and that relationships of family members, including parent-child relationships, can be undermined by parents’ stress [17]. The theoretical assumptions are substantiated by growing empirical evidence testing the impact of the COVID-19 pandemic on family functioning. For example, one study showed a decrease in family cohesion and an increase in family conflict which both were associated with child maladjustment [32]. Other studies also found that parents’ stress during the COVID-19 pandemic changed family relationships. Parents reported to have more arguments with their child and found it more difficult to enjoy parent-child interactions [12, 23]. As such, disruptions caused by the COVID-19 pandemic “get inside the family by altering interpersonal relations within the family” [6] (p. 632).

### Family processes as mediators

It is well established in the literature that acute and chronic stressors contribute to child adjustment problems. Knowledge about the mechanisms underlying these associations is crucial to inform policies and interventions aimed at supporting families. A central assumption of the FSM is that pathways to negative child outcomes are mediated by family processes [16, 33]. Importantly, empirical evidence suggests that family processes are malleable and programs can be effective in changing maladaptive family processes [34–36]. As such, testing family processes as mediators can provide useful information for optimizing and implementing policies and interventions.

However, empirical evidence regarding mediation pathways is mixed. For example, Mistry et al. [37] found that associations between children’s exposure to cumulative risk and their school readiness skills were (partially) mediated through family processes, while Shelleby et al. [38] found that family income led to higher levels of children’s conduct problems, observed through family processes. Yet, other studies did not find evidence for significant mediations via family processes, even though the individual paths included in the mediation model were significant [39]. An additional limitation of previous research is that mediations within the FSM framework was often tested using cross-sectional data [39]. However, cross-sectional mediator analysis can be biased in that indirect effects may be detected that do not hold in longitudinal analysis [40].

### The present study

This research reflects experiences of parents of young children (ages 0–8 years) living in the UAE during the COVID-19 pandemic. The overarching aim was to test the assumptions of the FSM in the pandemic context (Fig 1). We considered recent literature reviews to reflect the specifics of the COVID-19 pandemic when adapting the FSM which we based on frameworks of family wellbeing [6], relationship science [27], and life course perspectives [17].

The child-specific impacts of the COVID-19 pandemic depend on the developmental timing, with younger children being particularly vulnerable [17]. Based on the literature suggesting that COVID-19-related hardships and pressures may have similar effects on parents’ wellbeing as economic hardships and pressures, we expected to find associations with higher levels of parents’ stress. We also expected that higher levels of parents’ stress would show direct negative links with family relationships (i.e., higher spousal relationship problems and lower quality of parent-child relationships) and child wellbeing.

Because parents’ wellbeing “may serve as a funnel through which social disruptions due to COVID-19 infiltrate family functioning via changes to marital [and] parent-child […] relations” [6] (p. 632), we assumed that the associations between parents’ stress and child wellbeing would be mediated through family relationships. Specifically, we expected higher levels of parents’ stress to be related with lower quality family relationships which, in turn, would be linked to lower child wellbeing.

The COVID-19 pandemic was not a one-time shock but an ongoing stressor. We thus explored associations between COVID-19-related hardships and pressures, parents’ stress, family relationships, and child wellbeing over time. Because it is possible that time may either buffer or exacerbate the pandemic’s negative effects on families, we treated this aspect as an exploratory part of our analyses.

The study has several contributions to the literature. It extends research on the FSM beyond studies with school-aged children and adolescents [16] to younger children (birth to 8 years) who may be particularly vulnerable to developmental shocks resulting from the pandemic [17]. In addition, it extends research exploring the temporal dynamics between parent and child wellbeing during a period of unexpected and drastic societal change due to the COVID-19 pandemic [5, 9, 15]. By collecting data twice during the pandemic (t1, t2), the study helps to uncover immediate and lagged effects of the pandemic on parent and child wellbeing.

## Methods

### Participants

The Life During COVID project, a university-government collaboration exploring the experiences of families with young children during the COVID-19 pandemic, targeted parents of children aged 0–8 years in the Emirate of Abu Dhabi, UAE. After cases of COVID-19 started to rise in the UAE in February 2020, the government mandated people to avoid family gatherings and social events to reduce the risk of infections, a restriction particularly challenging within the cultural context of the UAE, where an important cultural pillar is close social bonds with the immediate and extended family [41, 42]. In early March 2020, businesses, places of worship, daycare centers, and schools were closed nationwide.

Initially, school closures were planned to last four weeks, but facilities remained closed for the remaining months of the academic year with distance learning implemented. Schools reopened in September 2020, but throughout the 2020–21 academic year, they were closed several times as cases climbed. Similarly, work-from-home policies were implemented to reduce the spread of the virus, forcing employees from various sectors to work remotely.

At the time of recruitment for the Life During COVID project from which we draw the data, in-person human subject research was not permitted due to the COVID-19 situation in the country. Thus, data was collected through an online survey. The study protocol was reviewed and approved by the Institutional Review Board of the authors’ university (protocol HRPP-2020-65). This study was not preregistered. Prior to completing the survey, all participants provided informed consent. In Fall 2020 (between September-November), the survey link was disseminated on social media (Facebook and Twitter), through short message service (SMS), and via a panel management service. The survey link dissemination strategy was similar to other studies conducted with parents during the pandemic (e.g., [12]). One recruitment goal of the present study was to particularly target UAE citizens who make up approx. 19% of the population in the Emirate of Abu Dhabi [43]. The government partner facilitated a targeted recruitment to reach that group of the population.

Initially, 5,165 survey link clicks were registered; 3,109 (60.2%) did not complete more than the first survey block with demographic questions. Entries were further checked for inclusion criteria (parents of children aged 0–8 years; residents in the Emirate of Abu Dhabi), which were not met by 316 respondents. Furthermore, 71 duplicates were identified and deleted. Disqualified entries (*n* = 3,496, 67.7% of all clicks) were removed.

The resulting sample included 1,669 participants in Fall 2020 (t1). The majority of participants (*n* = 1,096, 65.7%) were from countries other than the UAE; the remaining participants were UAE citizens (*n* = 573, 34.3%). While the proportion of UAE citizens in the sample was larger than that of the UAE’s total population (11.6%) [44], the sample reflects diversity of the population in the Emirate of Abu Dhabi. In an attempt to verify participants, we estimated their age at the child’s birth. Taking local traditions and customs into account (such as early marriage), no unreasonable responses were found. Participants were re-contacted for a follow-up survey between February-April 2021 (t2), when 1,144 survey link clicks were registered; 282 (24.7%) did not complete more than the first few demographic questions. These entries were again deleted. After removing cases that could not be merged with responses from the first data collection (9%), the final sample included 783 parents who completed the survey twice. Post-hoc power analysis for structural equation modeling yielded a minimum sample size of 589 participants (using the following assumptions: effect size = 0.2; statistical power level = 0.8; probability level = 0.05). Of these parents, 96 completed the survey for two children in the same 0–8 age range. We verified that this subsample was representative of the full sample. A series of *t*-tests showed that the subsample, for which data about two children in the same family was available, was a close representation of the full sample. There was no evidence that the two samples were different in terms of parent age, being a UAE national or not, and region of residence (S1 Table).

The mean age of parents (75% female) was 34.61 years (*SD* = 6.05) (see Table 1 for demographic details). Reflecting the diversity of the population in the UAE, most parents (69%) were from other countries (including Egypt, India, Jordan, Pakistan, the Philippines, Syria, and the United Kingdom); 31% were UAE citizens. Half of the sample resided in the city of Abu Dhabi (urban region) and half in suburban and rural regions which is representative of the general population in the Emirate of Abu Dhabi where 49.9% of the population live in the city of Abu Dhabi [45]. In terms of education, 44% of the sample held a bachelor’s degree or higher.

**Table 1:** Demographic information about the sample.

Full sample (*n* = 783): Information is available for one child at both t1 and t2					
	Mean	*SD*	Minimum	Median	Maximum
Parent age (in years)	34.61	6.05	21.00	34.00	59.00
Nationality: UAE nationals	31%	-	-	-	-
Education: BA and higher	44%	-	-	-	-
Child age (in months)	47.71	28.86	0.00	40.50	96.00
Child sex: male	57%	-	-	-	-
Subsample (*n* = 96): Information is available for two children in the family at t1					
	Mean	*SD*	Minimum	Median	Maximum
Parent age (in years)	34.55	5.69	21.00	36.00	48.00
Nationality: UAE nationals	38%	-	-	-	-
Education: BA and higher	53%	-	-	-	-
Child age (in months): Child 1	58.41	27.24	2.00	58.41	96.00
Child 2	42.36	27.42	1.00	45.50	96.00
Child sex: male Child 1	67%	-	-	-	-
Child 2	50%	-	-	-	-

### Measures

The survey was available in both Arabic and English and used Qualtrics. Arabic is the official language in the UAE; English is used in conjunction with Arabic in most sectors. The survey was translated and back-translated by native Arabic speakers who were fluent in English to ensure the quality of the translation and consistency of meaning. The same survey questions were asked at both measurements (t1 and t2). The first block focused on demographic information of the family, COVID-related indicators of hardships and pressures, parents’ stress, and spousal relationships problems. The second part asked about the wellbeing of their child and parents’ perceived quality of the relationship with them. If parents indicated that they had two children aged 0–8 years, they received a third part of the survey that asked questions regarding the second child’s wellbeing and their relationship quality with that child. In the demographic part of the survey, parents were asked to provide the name(s) of their child(ren). The questions regarding child 1 and child 2 were personalized and used the name for that child to make it easier for the parent to respond to these questions for each specific child.

#### COVID-19-related indicators of hardship and pressure

Parents were asked to indicate the extent to which they expect their family to experience economic pressure due to the COVID-19 pandemic, using the following item: *In the next few months*, *how likely is it that your family situation will get worse due to economic consequences of Coronavirus*? [46]. Parents rated the item using a 0–100 range to indicate the perceived likelihood of the statement becoming true (0 = *exceptionally unlikely* to 100 = *all but certain*); higher values reflected greater perceived economic pressure. Two yes-no questions measured whether parents, or someone in their household, lost their job due to the COVID-19 pandemic (“*Did you personally lose your job because of Coronavirus*?” and “*Did someone in your household lose their job because of Coronavirus*?”). A dummy-coded variable was created. If participants responded “no” to both questions, they were given a score of zero. All other participants who either personally lost their job because of the pandemic or had someone in their household who lost their job, received a score of 1.

Parents also reported the perceived health risk of COVID-19 for themselves and their family using the following two items: *In the next few months*, *how likely is it that you (someone in your family) will get infected with Coronavirus*? [46]. The same 0–100 range was used. The two questions were averaged into a single variable (t1: *r* = 0.69, *p*<0.001; t2: *r* = 0.73, *p*<0.001), with higher values reflecting greater perceived health risk of COVID-19.

#### Parents’ stress

Parents were asked about their perceived stress related to childrearing, using the items from the Fragile Families and Child Wellbeing Study [47]. Participants rated their agreement with these statements on a 4-point scale ranging from 1 (*strongly disagree*) to 4 (*strongly agree*). Higher values reflected higher levels of perceived stress. Internal consistency was good in the present sample (t1: α = .77; t2: α = .86).

#### Spousal relationship

Three items from the Parental Distress subscale of the Parenting Stress Index [48] measured participants’ relationship quality with their spouse. Parents rated the items on a 5-point scale (1 = *strongly disagree* to 5 = *strongly agree*). Higher values reflected perceived lower quality of the spousal relationship (i.e., higher levels of spousal relationship problems). Internal consistency was good in the sample (t1: α = .79; t2: α = .87).

#### Parent-child relationship

One item–*During the past 30 days*…*—I am satisfied with the amount of quality time spent with [child name]*.–was used as a proxy to assess parents’ perception of the relationship with their child. Parents rated the quality of the time spent with their child on a 5-point scale (1 = *strongly disagree* to 5 = *strongly agree*), with higher values reflecting higher levels of parent satisfaction with the relationship with their child. Parents who completed the survey for two children were asked to respond to the question for each child.

#### Child wellbeing

Parents were asked to rate the following aspects of their child’s wellbeing: health (general), physical health, sleep quality, social activities, quality of life, and mental health [49]. The same question–*In general*, *how would you rate…*?–was used for each category (*In general*, *how would you rate [child name] quality of life*?). Parents rated the items on a 5-point scale, coded 1 (*poor*), 2 (*fair*), 3 (*good*), 4 (*very good*), and 5 (*excellent*). A child wellbeing index was formed. Higher values reflected higher levels of child wellbeing. The internal consistency of the index was good (t1: α = .88; t2: α = .92). Parents who completed the survey for two children were asked to respond to these questions for each child.

#### Demographic and other control variables

Parents provided demographic background information, including their age, level of education, nationality (coded as: UAE national or other country of origin), and child age and sex. In several studies, parents reported decreased levels of their child’s physical activity during the pandemic [11, 50, 51]. Importantly, reduced physical activity has been associated with poorer mental health [52]. Therefore, we included child physical activity as control variable. Parents were asked to rate two items (*How often does [child name] usually play actively in the yard or area outside your home*? and *How often does [child name] usually play actively in outdoor areas*, *like parks*, *pools*, *and playgrounds*, *near your home*?) on a 5-point scale, coded 1 (*almost never*), 2 (*1–2 times per week*), 3 (*3–4 times per week*), 4 (*5–6 times per week*), and 5 (*everyday*). The items were taken from the Hop-Up questionnaire [53]. For further analyses, the two items were averaged (t1: *r* = .47, *p* < .001; t2: *r* = .44, *p* < .001), with higher values reflecting higher frequency of physical activity.

### Analytical strategy

We first created latent variables for the constructs that were measured with three or more items (such as parents’ stress, spousal relationship problems, and child wellbeing) using confirmatory factor analyses (CFA). When necessary, modifications were introduced to reach the best fitting model. Because constructs were measured at two time points, multi-wave CFAs were used to test their measurement invariance over time at three levels: configural, metric, and scalar. Configural invariance assumed the same scale structure over the two waves without restricting factor loadings of the scale items. Metric invariance assumed the same scale structure over time by fixing the factor loadings of the items across the two waves.

In addition to these two conditions, scalar invariance additionally fixed the intercepts of the scales’ items across the two waves. Change in comparative fit index across the configural, metric, and scalar models (>±0.01 considered the cutoff) was used to determine measurement invariance [54]. In addition, CFA model fit was evaluated using the root mean square error of approximation (RMSEA; < 0.08), comparative fit index (CFI; > 0.90), the Tucker–Lewis index (TLI; > 0.90), and standardized root mean residual (SRMR; < 0.08) [55]. The results are presented in S2 Table.

Next, structural equation modeling (SEM) was then used to test the assumptions of the FSM in the context of the COVID-19 pandemic. The SEM analyses were conducted in RStudio (version 1.4.1106), using the following variables: COVID-19-related indicators of hardships and pressure, parents’ stress, spousal relationship problems, parent-child relationship, and child wellbeing. Control variables (t1: parent age [in years], level of education [binary; 1: BA degree or higher, 0: otherwise], nationality [binary; 1: UAE national, 0: otherwise], child age [in months], child sex [binary; 1: boy, 0: girl], and physical activity) were included. The model estimated associations concurrently (t1, t2) for one child in a family (the same child at t1 and t2) (Fig 1). In addition, autoregressive paths (such as the path between parents’ stress at t1 and parents’ stress at t2) and cross-lagged paths (for instance, the path between parents’ stress at t1 and spousal relationship problems at t2) were estimated to explore relationships over time (t1-t2).

In a supplemental analysis, a second SEM estimated concurrent (t1) associations between the variables of interest for families with data reported from two children (S1 Fig). The goal of the supplemental analysis was to assess whether similar associations are evident for two children of similar age (0–8 years) in the same family, and in particular, to examine child-specific parent-child relationship quality amid COVID-19 stressors. The results are presented in S2 Fig and S3 Table.

SEM models were fitted with both maximum likelihood parameter estimates (MLM) and maximum likelihood with robust standard errors (MLR) whereby the latter results are reported here (MLM results can be found in the supplementary information, S3 and S4 Tables). For both models, tests of direct and indirect effects were run. To test the full mediation effect, an inferential test of the entire specific indirect effect was conducted. More specifically, we tested whether the indirect effect of parents’ stress on child wellbeing via (a) spousal relationship problems or (b) parent-child relationship was significant, and (c) whether the overall indirect effect of parent stress on child wellbeing was significant. For each predictor-to-outcome effect, we used the lavaan package [56] to first compute the direct effect and then the indirect effect (along with standard errors and significance tests). Standard errors were computed using the delta method (producing what is known as the Sobel test for indirect effects). The standardized regression coefficients were used as effect size measures, with β ≈ 0.10 indicating a small effect, a β of ≈ 0.20 a typical-sized effect, and β>0.30 indicating a large effect [57]. In addition, we reported the R-squared for each latent variable in the direct effects models to estimate the proportion of variance explained by all predictors in the model.

Data preparation and analyses were performed using Stata and R. Missing data were handled by multiple imputation using the MICE package in R [58]. Predictive mean matching algorithm was selected for the multiple imputation as it is a versatile method that allows discrete variables in the data and the resulting imputed data are realistic, given that they fall within the range of the observed variables [59]. Data and code for all analyses are available (https://github.com/rali314/family_stress_during_covid_19_pandemic).

## Results

### COVID-19-related hardships and pressures and parents’ stress

**Fig 2:**
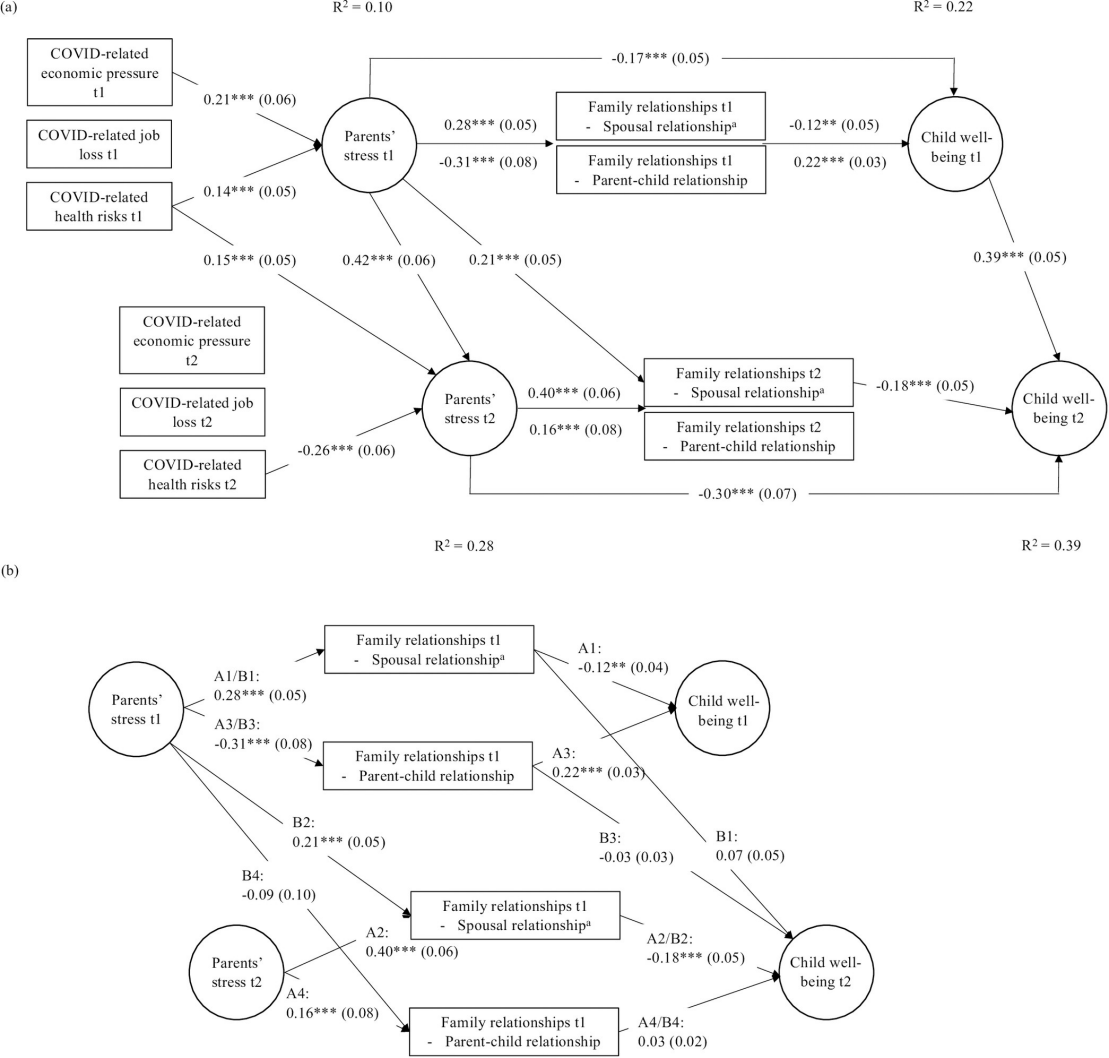
Parameter estimates from SEM testing assumptions of the FSM. (a) Associations between COVID-19-related hardships and pressures on parents’ stress as well as direct associations between parents’ stress (and family relationships, respectively) and child wellbeing cross-sectional at t1 (t2, respectively) and over time (t1-t2). (b) Cross-sectional (A paths) and lagged (B paths) mediation pathways. The model controlled for parent age, level of education, and nationality, and child sex, age, and physical activity. The analysis reported here used the MLR estimator. Model fit: CFI = 0.906; RMSEA = 0.048; SRMR = 0.062. * *p* ≤ 0.05; ** *p* ≤ 0.01; *** *p* ≤ 0.001. ^a^ Higher values reflect higher levels of spousal relationship problems.

The first SEM model estimated associations concurrently and over time for one child in a family, using the full sample (*N* = 783) (Fig 1; S4 Table shows the correlation between variables). Parents’ perceived COVID-19-related health risks were significantly correlated to parents’ stress, however, the direction of association differed across time (Fig 2A and S5 Table). At t1, fears of getting infected with the coronavirus were positively related with parents’ stress (*β* = 0.14, *p*<0.01). The association was negative at t2 (*β* = -0.256*p*<0.001), but positive over time (t1-t2) (*β* = 0.15, *p*<0.01). No significant associations were found between COVID-19-related job loss and parents’ stress, neither concurrently at t1 (*β* = 0.05, n.s.) or t2 (*β* = 0.03, n.s.) nor over time (t1-t2) (*β* = 0.06, n.s.). COVID-19-related economic pressure was significantly associated with parents’ stress at t1 (*β* = 0.21, *p*<0.01), but not at t2 (*β* = 0.00, n.s.) or over time (t1-t2) (*β* = -0.03, n.s.).

### Parents’ stress and family relationships

Parents’ stress was positively and significantly associated with spousal relationship problems at t1 and t2 (t1: *β* = 0.28, *p*<0.01; t2: *β* = 0.40, *p*<0.001) and over time (t1-t2) (*β* = 0.21, *p*<0.001) (Fig 2B and S5 Table). Higher levels of parents’ stress were related to increased relationship problems between the caregiving partners. Similarly, higher levels of parents’ stress were related to lower perceived quality of the parent-child relationship. Associations were significant and negative at t1 (*β* = -0.31, *p*<0.001) but positive at t2 (*β* = 0.16, *p*<0.001). The association between parents’ stress at t1 and parent-child relationship quality at t2 was not significant (*β* = -0.09, n.s.).

### Direct and indirect associations between parents’ stress and child wellbeing over time

The direct association between parents’ stress and child wellbeing was significant and negative at t1 (*β* = -0.17, *p*<0.01) and t2 (*β* = -0.30, *p*<0.001) (Fig 2A and S5 Table). No significant association was found between parents’ stress at t1 and child wellbeing at t2 (*β* = -0.05, n.s.). To explore indirect associations between parents’ stress and child wellbeing, we first examined the individual direct paths between the mediators (spousal relationship and parent-child relationship) and child wellbeing (Fig 2A and S5 Table). The association between spousal relationship problems and child wellbeing was significant and negative at t1 (*β* = -0.12, *p*<0.01) and t2 (*β* = -0.18, *p*<0.05), but the association between spousal relationship problems at t1 and child wellbeing at t2 was not significant (*β* = 0.07, n.s.). The association between parent-child relationship and child wellbeing was significant and positive at t1 (*β* = 0.22, *p*<0.001). Neither the association at t2 (*β* = 0.03, n.s.) nor the association over time (parent-child relationship at t1 and child wellbeing at t2; *β* = -0.03, n.s.) were significant. A total of eight mediation pathways from parents’ stress to child wellbeing were tested: four cross-sectional mediations (“A” paths; mediated by family relationships at t1 and t2) and four lagged mediations (“B” paths) exploring mediation pathways over time (Fig 2B and S5 Table). For the latter, we tested two conditions: (a) effect of family relationships at t1 on child wellbeing at t2 (paths B1 and B3) and (b) effect from parents’ stress at t1 on family relationships at t2, which were related to child wellbeing at t2 (paths B2 and B4).

Regarding the cross-sectional mediations (Fig 2B), we found evidence for significant, albeit small indirect associations between parents’ stress and child wellbeing via spousal relationship problems at t1 (path A1: *β* = -0.03, *p*<0.05) and t2 (path A2: *β* = -0.07, *p*<0.05), and via parent-child relationship at t1 (path A3: *β* = -0.07, *p*<0.001). The indirect association via parent-child relationship at t2 was not significant (path A4: *β* = 0.00, n.s.). Only one of the cross-sectional mediations was replicated longitudinally. We found a similar small significant indirect association between parents’ stress at t1 and child wellbeing at t2 via spousal relationship problems at t2 (second condition/path B2: *β* = -0.05, *p*<0.05) but not via spousal relationship problems at t1 (first condition/path B1: *β* = 0.02, n.s.). None of the lagged mediations via parent-child relationship was significant (via parent-child relationship at t1/path B3: *β* = 0.01, n.s.; via parent-child relationship at t2/path B4: *β* = 0.00, n.s.).

## Discussion

The impact of the COVID-19 pandemic on parent and child wellbeing has been covered extensively in the literature but with limited focus on the mechanisms through which pandemic-related stressors may affect these dynamics. The current study extends the literature on family functioning during the COVID-19 pandemic by testing patterns of associations with pandemic stressors, family relationships, and child adjustment. Our findings suggest that the links between pandemic-related hardships and pressures and family processes were like those between economic stressors and family processes previously researched [16]. Pandemic-related stressors contributed to higher stress among parents early in the pandemic and parents’ stress resulted in lower parent-reported child wellbeing at various times during the pandemic. Family relationships mediated the association between parents’ stress and child wellbeing. The present study contributes to our understanding on how disruptions might get inside the family [5, 14], the strength and direction of associations (concurrently and over time), and the timing of mechanisms that impact family processes.

In line with our hypothesis, the present study found that COVID-19-related hardships and pressures were associated with higher levels of parents’ stress, suggesting that the pandemic, like economic hardships, creates conditions that may result in stress reactions among parents [6, 22]. The association, however, was only significant at t1 (Fall 2020) but not at t2 (Spring 2021). One explanation is that, as the pandemic continued, parents might have adapted to and seemed to cope well COVID-19-related stressors [9]. For example, parents could have coped with COVID-19-related hardships and pressures by minimizing catastrophic thinking [6]. In addition, vaccinations against COVID-19 became available to the public between the two waves of data collection, which might have influenced parents’ views of the COVID-19-related hardships and pressures in a way that reduced stress. However, the results of the present study might be specific to the population studied, that is, families in an affluent country who likely experienced less economic stressors before the pandemic and, consequently, might have been less vulnerable to pandemic-related hardships and pressures.

Additionally, an interesting pattern was observed for perceived health risks. While at t1 and over time (t1-t2), the association suggested that higher health risks were associated with higher levels of parents’ stress, at t2, the association was negative, suggesting that higher health risks were associated with lower levels of parenting stress. We can only speculate about possible reasons for this unexpected finding. In the UAE, the strict and mandatory public health measures (such as wearing masks in public and social distancing) might have helped parents to cope with COVID-related health risks so that, despite a spike in COVID infections during the period of the second data collection, stress reactions were less likely. The finding might also reflect a more general pattern, such that pandemic conditions and health risks did not produce long-term distress [60]. In fact, we found that levels of psychological distress returned to pre-pandemic levels after increasing early in the pandemic. But the study also highlights that some individuals experienced more lasting risks to their wellbeing [60].

Consistent with existing literature from before [39, 61, 62] and during the pandemic [12, 23, 27], and in line with our hypothesis, we found that parents’ stress was associated with more problematic relationships between the parenting partners, and between the parent and child. We also found that, overall, parents’ stress and problematic family relationships were associated with lower levels of parent-reported child wellbeing, thus supporting our hypothesis. These findings suggest that the wellbeing of one family member can impact family processes and relationships that may put the wellbeing of the entire family at risk [6]. This is problematic, because previous research found that the quality of family relationships acts as a buffer against the negative effects of (natural) disasters and adversities on children’s wellbeing [5, 63]. It is possible that the specific restrictions due to the COVID-19 pandemic, such as stay-at-home and work-from-home policies, added to family stress processes [9]. More specifically, previous research found that risks for parent and child wellbeing were particularly high at the peak of COVID-19 restrictions [5]. Given that the pandemic environment changed quickly (as expected in a public health crisis with its ups and downs in infection rates), it will be important to consider the temporal dynamics between parent and child wellbeing in policy and practice efforts to ensure that families are optimally supported during pandemic emergencies.

Furthermore, we assumed that the associations between parents’ stress and child wellbeing would be mediated through family relationships. The hypothesis could be partially confirmed because the results depended on whether mediations were tested with cross-sectional or longitudinal data. At both time points (cross-sectional data), significant (albeit very small) indirect effects between parents’ stress and child wellbeing via family relationships were identified. These findings are in line with assumptions of the FSM [16] and the framework of family wellbeing [6], suggesting that one mechanism underlying the associations between COVID-19-related disruptions and child adjustment problems is altering interpersonal relations within the family. It is possible that the stay-at-home and work-from-home policies, and the uncertainty about their end, disrupted communicative and organizational (routines, rituals, and rules) processes in families leading to more conflict in family relationships [6]. Thus, a focus of interventions should be on restoring close relationships within the family to help family members (and the entire family) cope with the adverse experiences due to the pandemic [6].

However, the detected mediations did not replicate when lagged pathways were considered, thus limiting conclusions regarding the stability of mechanisms over time. In addition, other studies from during the pandemic did not find evidence that “the amount of stress that parents and adolescents experienced was […] directly linked to changes in the quality of their relationship” [9] (p. 1619). The divergent findings might be due to differences in patterns of parent-child interactions at different developmental levels more generally. In our study, we reported experiences of families with young children (0–8 years), whereas Donker et al. [9] included families with adolescents. During adolescence, negative interactions “are thought to have an important function in the development of a more equal relationship between parents and adolescents” (Branje, 2018; Branje et al., 2012; cited in [9], p. 1619). Negative interactions during the early childhood years do not serve such a function and have been linked with negative outcomes, such as increases in children’s negative emotions [64], sleep problems [65], or internalizing problems [66]. More research investigating the mechanisms underlying associations between stressors and child adjustment is needed, particularly longitudinal research, as our results point to the importance of investigating family processes (stress and resilience) in longitudinal analysis to detect whether similar or different mechanisms may be at play at different points in a family’s history.

### Limitations

Several additional limitations need to be considered. Most importantly, variables were not assessed before the pandemic and, as such, we cannot rule out that pre-existing family vulnerabilities such as marginalization, mental health conditions, or family relational dysfunction [6], contributed to family processes and how pandemic adversities were navigated. Methodological challenges, in particular potential sampling bias and shared method bias, call for caution in interpreting the findings. The reliance on an online convenience sample with self-selected participants could have led to biased estimates, thus limiting the generalizability of findings [67]. Similar to other studies during the COVID-19 pandemic that used online surveys [12, 68], we do not know if and in what ways participants in our study differ from parents who chose not to participate in the study. As in prior studies [14], some of the observed associations might have been because the study relied on parents as the only informants. Parents reported information on their own psychosocial functioning, the functioning of family relationships, and their child’s psychosocial functioning. In-person human subject research was restricted during the time of data collection, limiting our ability to include other sources of data. Future research should include multiple sources of data, such as data from children and other informants (like teachers), and observations of family interactions and parent and child behaviors and outcomes.

In addition, one item assessing child wellbeing (social activities) did not show sufficient consistency with the construct across the two measurements and was therefore dropped from the analyses. Prolonged stay-at-home policies limited children’s social activities and parents might have had few or no opportunities to observe their child engaging in such activities as the pandemic continued. Thus, they might have used a different frame of reference when responding to this item. Furthermore, most respondents were mothers (75%). Caregiving has fallen disproportionally to mothers during the pandemic [69]. However, because our interest was not on levels of wellbeing but rather on patterns of associations between COVID-19-related disruptions and wellbeing, we think the potential bias introduced to the results is minimal.

### Conclusion

The consistency of our findings with earlier tests of the FSM suggests family-based pathways that may alleviate or exacerbate child adjustment problems as consequences of adverse events. Although stressors might have been different during the COVID-19 pandemic as compared to pre-pandemic economic stressors, our findings suggest that the disruptions due to COVID-19 put parents at risk for experiencing stress reactions that may reduce caregivers’ ability to protect, support, and promote children’s wellbeing. This study contributes to understanding the mechanisms through which COVID-19-pandemic stressors may impact family processes and children’s development. As the pandemic continues, it will be important to develop and implement interventions that support families in maintaining or regaining a positive family climate that enables supportive relationships to prevent negative consequences on wellbeing.

## Supporting information

S1 Fig Conceptual model for associations between COVID-19-related hardships and pressures, parents’ stress, family relationships, and child wellbeing in two-child families. (TIF)

S2 Fig Parameter estimates from SEM testing assumptions of the FSM in two-child families. (a) Associations between COVID-19-related hardships and pressures and parents’ stress, as well as direct associations between parents’ stress (and family relationships, respectively) and child wellbeing for two children within the same family. (b) Cross-sectional mediation pathways for child 1 and child 2 (“A” paths via spousal relationship problems; “B” paths via parent-child relationship). The model controlled for parent age, level of education, and nationality, and child sex, age, and physical activity. The analysis reported here used the MLR estimator. Model fit: CFI = 0.911; RMSEA = 0.062; SRMR = 0.075. * *p* ≤ 0.05; ** *p* ≤ 0.01; *** *p* ≤ 0.001. ^a^ Higher values reflect higher levels of spousal relationship problems.
(TIF)

S1 Table Results of *t*-tests comparing the full sample and subsample with regard to demographic characteristics of the parents. (DOCX)

S2 Table Tests of measurement invariance. (DOCX)

S3 Table Testing assumptions of the FSM in two-child families. (DOCX)

S4 Table Correlation between variables (full sample, n = 783). (DOCX)

S5 Table Results of the structural equation modeling testing assumptions of the FSM (first row: MLR estimation; second row: MLM estimation). (DOCX)
