# Supplementary figures and images for: COVID-19’s shadow on families: A structural equation model of parental stress, family relationships, and child wellbeing
Source: PLoS One. 2023 Oct 12;18(10):e0292292. doi: 10.1371/journal.pone.0292292 (PMC10569562; doi:10.1371/journal.pone.0292292)

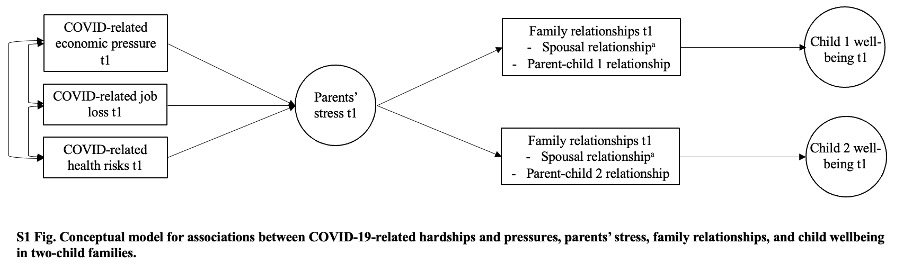

Supplement: S1 Fig — (TIF) [file pone.0292292.s001.tif]

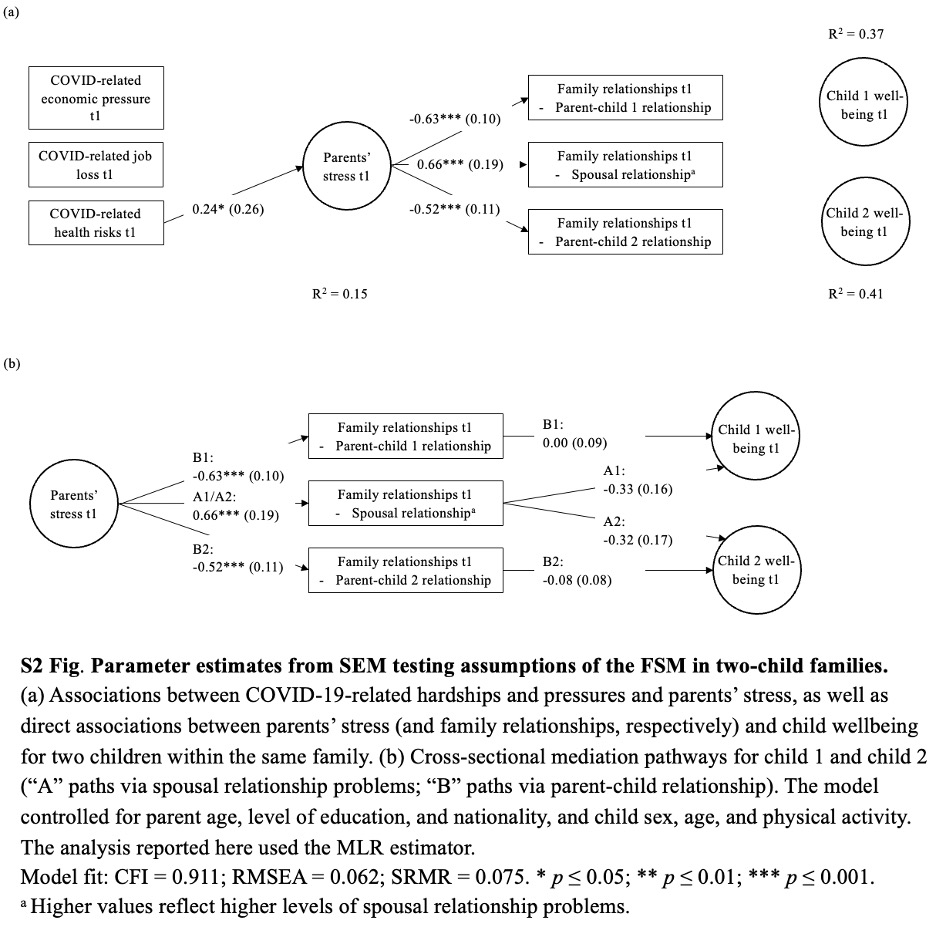

Supplement: S2 Fig — (a) Associations between COVID-19-related hardships and pressures and parents’ stress, as well as direct associations between parents’ stress (and family relationships, respectively) and child wellbeing for two children within the same family. (b) Cross-sectional mediation pathways for child 1 and child 2 (“A” paths via spousal relationship problems; “B” paths via parent-child relationship). The model controlled for parent age, level of education, and nationality, and child sex, age, and physical activity. The analysis reported here used the MLR estimator. Model fit: CFI = 0.911; RMSEA = 0.062; SRMR = 0.075. * p ≤ 0.05; ** p ≤ 0.01; *** p ≤ 0.001. a Higher values reflect higher levels of spousal relationship problems. (TIF) [file pone.0292292.s002.tif]
