# Supplementary material for: COVID-19’s shadow on families: A structural equation model of parental stress, family relationships, and child wellbeing
Source: PLoS One. 2023 Oct 12;18(10):e0292292. doi: 10.1371/journal.pone.0292292 (PMC10569562; doi:10.1371/journal.pone.0292292)
Supplement: S1 Table — (DOCX) [file pone.0292292.s003.docx]

**S1 Table. Results t-tests comparing the full sample and subsample with regard to demographic characteristics of the parents.**

| Variable | Full sample (*n*=783) | Subsample (*n*=96) | *p*-value |
| --- | --- | --- | --- |
| Parent age | 34.61 | 34.55 | 0.86 |
| Parent’s nationality: UAE nationals | 31% | 38% | 0.20 |
| Parent’s education: BA and higher | 44% | 53% | 0.12 |
