# Supplementary material for: COVID-19’s shadow on families: A structural equation model of parental stress, family relationships, and child wellbeing
Source: PLoS One. 2023 Oct 12;18(10):e0292292. doi: 10.1371/journal.pone.0292292 (PMC10569562; doi:10.1371/journal.pone.0292292)
Supplement: S2 Table — (DOCX) [file pone.0292292.s004.docx]

**Tests of measurement invariance**

CFAs were used to test the structure of each latent construct (parents’ stress, spousal relationship, and child wellbeing). Results indicated satisfactory latent variable structure for parents’ stress and spousal relationship (S2a Table), and scalar invariance was achieved for both constructs without additional modifications (S2b Table). For child wellbeing, the CFA resulted in a satisfactory model including all items of child wellbeing (S2a Table). However, scalar invariance could not be established (delta CFI > 0.01; S2b Table). Examination of the items revealed that the item asking about the child’s social activities did not show sufficient consistency over time. Therefore, the item was dropped, and measurement invariance could be established at all three levels (S2b Table). The modified version of the child wellbeing index was used for all subsequent analyses. Measurement invariance was also tested across in two-child families using the same modified specifications, and the results indicated that measurement invariance could be achieved at all three levels (S2b Table).

**S2a Table. Confirmatory factor analyses: Model fit and reliability and validity assessment.**

|  |  | t1 | | | | | t2 | | | | |
| --- | --- | --- | --- | --- | --- | --- | --- | --- | --- | --- | --- |
| Factors | Items | Factor Loading^a^ | Cronbach’s Alpha | CFI | RMSEA | SRMR | Factor Loading^a^ | Cronbach’s Alpha | CFI | RMSEA | SRMR |
| Parents’ stress | Being a parent is harder than expected. | 0.71 | 0.76 | 0.99 | 0.04 | 0.01 | 0.79 | 0.85 | 0.99 | 0.07 | 0.01 |
|  | I feel trapped by my responsibilities as a parent. | 0.85 |  |  |  |  | 0.82 |  |  |  |  |
|  | Taking care of my child(ren) is much more work than pleasure. | 0.44 |  |  |  |  | 0.63 |  |  |  |  |
|  | I often feel tired, worn out, or exhausted from raising a family. | 0.63 |  |  |  |  | 0.83 |  |  |  |  |
| Spousal Relationship | My partner does not give me help as much as I expected. | 0.68 | 0.80 | 1.00 | 0.00 | 0.00 | 0.76 | 0.86 | 1.00 | 0.00 | 0.00 |
|  | Problems happened in my relationship with my partner more than I expected. | 0.74 |  |  |  |  | 0.86 |  |  |  |  |
|  | I and my partner no longer share together in doing many things. | 0.84 |  |  |  |  | 0.84 |  |  |  |  |
| Child Wellbeing | Sleep quality | 0.76 | 0.86 | 1.00 | 0.00 | 0.00 | 0.71 | 0.87 | 0.99 | 0.04 | 0.01 |
|  | Mental health | 0.83 |  |  |  |  | 0.78 |  |  |  |  |
|  | Physical health | 0.80 |  |  |  |  | 0.84 |  |  |  |  |
|  | Life quality | 0.66 |  |  |  |  | 0.63 |  |  |  |  |
|  | Social activities | 0.62 |  |  |  |  | 0.72 |  |  |  |  |

^a^ Standardized values are shown.

**S2b Table. Measurement invariance of parents’ stress, spousal relationship, and child wellbeing across time, and child wellbeing across children in two-child families.**

|  |  | CFI | ΔCFI |
| --- | --- | --- | --- |
| Parent stress |  |  |  |
|  | Configural model | 0.998 |  |
|  | Metric invariance | 0.989 | 0.009 |
|  | Scalar invariance | 0.989 | 0.000 |
| Spousal relationship | |  |  |
|  | Configural model | 1.000 |  |
|  | Metric invariance | 0.999 | 0.001 |
|  | Scalar invariance | 0.996 | 0.000 |
| Child wellbeing across time | |  |  |
|  | Configural model | 1.000 |  |
|  | Metric invariance | 0.977 | 0.001 |
|  | Scalar invariance | 0.980 | 0.019 |
| Child wellbeing across time - modified | |  |  |
|  | Configural model | 1.000 |  |
|  | Metric invariance | 0.999 | 0.001 |
|  | Scalar invariance | 0.997 | 0.002 |
| Child wellbeing across children in two-child families - modified | | | |
|  | Configural model | 0.989 |  |
|  | Metric invariance | 0.987 | 0.000 |
|  | Scalar invariance | 0.987 | 0.000 |
