# Supplementary material for: COVID-19’s shadow on families: A structural equation model of parental stress, family relationships, and child wellbeing
Source: PLoS One. 2023 Oct 12;18(10):e0292292. doi: 10.1371/journal.pone.0292292 (PMC10569562; doi:10.1371/journal.pone.0292292)
Supplement: S3 Table — (DOCX) [file pone.0292292.s005.docx]

**Testing Assumptions of the FSM in Two-Child Families**

The supplemental SEM model represented a subsample (*n*=96) from the larger sample where information was available from two children (age 0-8 years) within the same family. The model only included concurrent data from t1 (S3a Table shows the correlation between variables). The sample size of parents who reported data from two children at t2 dropped significantly (*n*=41) compared to t1 (*n*=96) and did not allow for analyzing the model over time. Results are shown in S2 Fig and S3b Table.

Direct associations are shown in S2a Fig. Parents’ perceived COVID-19-related health risks were positively associated with parents’ stress (*β*=0.24, *p*<.05). The association between COVID-19-related job loss and parents’ stress was not significant (*β*=0.22, n.s.). The associations between COVID-19-related job loss and parents’ stress (*β*=0.22, n.s.) and between COVID-19-related economic pressure and parents’ stress (*β*=0.11, n.s.) were not significant. Regarding associations between parents’ stress and family relationships, we found a significant positive association with spousal relationship problems (*β*=0.66, *p*<0.01). For both children, the association between parents’ stress and parent-child relationship quality was significant and negative (child 1: *β*=-0.63, *p*<0.001; child 2: *β*=-0.52, *p*<0.001). The associations between parent-child relationship quality and child wellbeing were not significant (child 1: *β*=0.00, n.s.; child 2: *β*=-0.08, n.s.). The associations between spousal relationship problems and child wellbeing were negative and not significant (child 1: *β*=-0.33, n.s.; child 2: *β*=-0.32, n.s.). No significant direct associations were found between parents’ stress and each child’s wellbeing (child 1: *β*=-0.22, n.s.; child 2: *β*=-0.37, n.s.).

Regarding indirect associations between parents’ stress and child wellbeing (S2b Fig), we tested four cross-sectional mediations (two for each child: one via spousal relationship problems [“A” paths], and one via parent-child relationship [“B” paths]). No significant indirect associations were found (via spousal relationship problems: child 1 [A1]: *β*=-0.22, n.s.; child 2 [A2]: *β*=-0.21, n.s.; via parent-child relationship: child 1 [B1]: *β*=0.00, n.s.; child 2 [B2]: *β*=0.04, n.s.).

**S3a Table. Correlation between variables in two-child families.**

|  | Mean | *SD* | Parents’ stress  t1 | Spousal relationship^a^ t1 | Parent-child 1 relationship  t1 | Child 1 wellbeing t1 | Parent-child 2 relationship t1 | Child 2 wellbeing t1 |
| --- | --- | --- | --- | --- | --- | --- | --- | --- |
| Parents’ stress t1 | 2.61 | 0.68 | -- |  |  |  |  |  |
| Spousal relationship^a^ t1 | 2.44 | 1.11 | 0.50 | -- |  |  |  |  |
| Parent-child 1 relationship t1 | 1.29 | 1.45 | -0.29 | -0.31 | -- |  |  |  |
| Child 1 wellbeing t1 | 3.71 | 0.89 | -0.36 | -0.32 | 0.66 | -- |  |  |
| Parent-child 2 relationship t1 | 1.38 | 1.39 | -0.26 | -0.29 | 0.95 | 0.64 | -- |  |
| Child 2 wellbeing t1 | 3.64 | 1.00 | -0.38 | -0.37 | 0.58 | 0.91 | 0.57 | -- |

^a^ Higher values reflect higher levels of spousal relationship problems.

**S3b Table. Results of the structural equation modeling testing assumptions of the FSM in two-child families (first row: MLR estimation; second row: MLM estimation).**

| *Effect* | *β (SE)* | *std β* | *z-value* | *95% CI* |
| --- | --- | --- | --- | --- |
| COVID-19 related economic pressure → Parents’ stress | 0.25 (0.28) | 0.11 | 0.90 | (-0.30,0.80) |
|  | 0.25 (0.26) | 0.11 | 0.99 | (-0.25,0.76) |
| COVID-19 related job loss → Parents’ stress | 0.62 (0.32) | 0.22 | 1.92 | (-0.01,1.25) |
|  | 0.62 (0.32) | 0.22 | 1.97* | (0,10, 0.24) |
| COVID-19 related health risks → Parents’ stress | 0.56 (0.26) | 0.24 | 2.18* | (0.06,1.07) |
|  | 0.56 (0.26) | 0.24 | 2.19* | (0.06,1.06) |
| **Child 1** |  |  |  |  |
| Predictors |  |  |  |  |
| Parents’ stress → Parent-child relationship | -0.70 (0.1) | -0.63 | -6.96*** | (-0.9,-0.51) |
|  | -0.70 (0.11) | -0.63 | -6.37*** | (-0.92,-0.49) |
| Spousal relationship^a^ → Child wellbeing | -0.28 (0.16) | -0.33 | -1.77 | (-0.58,0.03) |
|  | -0.28 (0.15) | -0.33 | -1.88 | (-0.56,0.01) |
| Parent-child relationship → Child wellbeing | 0.00 (0.09) | 0.00 | 0.02 | (-0.18,0.18) |
|  | 0.00 (0.08) | 0.00 | 0.02 | (-0.16,0.16) |
| Parents’ stress → Child wellbeing | -0.26 (0.22) | -0.22 | -1.17 | (-0.69,0.18) |
|  | -0.26 (0.21) | -0.22 | -1.23 | (-0.67,0.15) |
| Covariates |  |  |  |  |
| Nationality: UAE → Parent-child relationship | 0.54 (0.21) | 0.22 | 2.61** | (0.13,0.94) |
|  | 0.54 (0.22) | 0.22 | 2.48*** | (0.11,0.97) |
| Education: BA degree and higher → Parent-child relationship | 0.09 (0.23) | 0.04 | 0,40 | (-0.36,0.54) |
|  | 0.09 (0.21) | 0.04 | 0,44 | (-0.32,0.50) |
| Parent’s age (std) → Parent-child relationship | -0.81 (0.62) | -0.11 | -1,31 | (-2.03,0.40) |
|  | -0.81 (0.64) | -0.11 | -1,26 | (-2.07,0.45) |
| Male child → Parent-child relationship | -0.04 (0.14) | -0.01 | -0,26 | (-0.32,0.25) |
|  | -0.04 (0.13) | -0.01 | -0,29 | (-0.29,0.22) |
| Nationality: UAE → Child wellbeing | 1.12 (0.28) | 0.43 | 3.97*** | (0.57,1.67) |
|  | 1.12 (0.28) | 0.43 | 4.01*** | (0.57,1.66) |
| Education: BA degree and higher → Child wellbeing | 0.08 (0.27) | 0.03 | 0.29 | (-0.44,0.60) |
|  | 0.08 (0.24) | 0.03 | 0.33 | (-0.39,0.55) |
| Male child → Child wellbeing | 0.14 (0.13) | 0.05 | 1.09 | (-0.11,0.39) |
|  | 0.14 (0.11) | 0.05 | 1.21 | (-0.09,0.36) |
| Child’s age (std) → Child wellbeing | -0.22 (0.13) | -0.08 | -1.67 | (-0.47,0.04) |
|  | -0.22 (0.15) | -0.08 | -1.48 | (-0.51,0.07) |
| Child’s outdoor activity → Child wellbeing | 0.17 (0.10) | 0.13 | 1.74 | (-0.02,0.36) |
|  | 0.17 (0.08) | 0.13 | 2.02* | (0.00,0.33) |
| **Child 2** |  |  |  |  |
| Predictors |  |  |  |  |
| Parents’ stress → Parent-child relationship | -0.58 (0.11) | -0.52 | -5.15*** | (-0.8,-0.36) |
|  | -0.58 (0.12) | -0.52 | -5.04*** | (-0.81,-0.36) |
| Spousal relationship^a^ → Child wellbeing | -0.27 (0.17) | -0.32 | -1.62 | (-0.61,0.06) |
|  | -0.27 (0.15) | -0.32 | -1.85 | (-0.56,0.02) |
| Parent-child relationship → Child wellbeing | -0.09 (0.08) | -0.08 | -1.08 | (-0.25,0.07) |
|  | -0.09 (0.07) | -0.08 | -1.21 | (-0.23,0.05) |
| Parents’ stress → Child wellbeing | -0.45 (0.24) | -0.37 | -1.86 | (-0.92,0.03) |
|  | -0.45 (0.21) | -0.37 | -2.14* | (-0.86,-0.04) |
| Covariates |  |  |  |  |
| Nationality: UAE → Parent-child relationship | 0.36 (0.22) | 0.14 | 1.66 | (-0.07,0.78) |
|  | 0.36 (0.23) | 0.14 | 1.53 | (-0.10,0.81) |
| Education: BA degree and higher → Parent-child relationship | -0.07 (0.22) | -0.03 | -0.30 | (-0.50,0.37) |
|  | -0.07 (0.22) | -0.03 | -0.30 | (-0.50,0.36) |
| Parent’s age (std) → Parent-child relationship | -1.04 (0.63) | -0.14 | -1.65 | (-2.27,0.20) |
|  | -1.04 (0.69) | -0.14 | -1.51 | (-2.39,0.31) |
| Male child → Parent-child relationship | -0.01 (0.12) | 0.00 | -0.08 | (-0.24,0.22) |
|  | -0.01 (0.13) | 0.00 | -0.07 | (-0.27,0.25) |
| Nationality: UAE → Child wellbeing | 0.92 (0.28) | 0.34 | 3.29*** | (0.37,1.47) |
|  | 0.92 (0.27) | 0.34 | 3.39*** | (0.39,1.45) |
| Education: BA degree and higher → Child wellbeing | 0.17 (0.27) | 0.06 | 0.63 | (-0.36,0.69) |
|  | 0.17 (0.24) | 0.06 | 0.70 | (-0.30,0.64) |
| Male child → Child wellbeing | 0.08 (0.11) | 0.03 | 0.72 | (-0.13,0.29) |
|  | 0.08 (0.11) | 0.03 | 0.70 | (-0.14,0.29) |
| Child’s age (std) → Child wellbeing | -0.37 (0.15) | -0.14 | -2.42** | (-0.67,-0.07) |
|  | -0.37 (0.15) | -0.14 | -2.53** | (-0.66,-0.08) |
| Child's outdoor activity → Child wellbeing | 0.17 (0.10) | 0.13 | 1.81 | (-0.01,0.36) |
|  | 0.17 (0.08) | 0.13 | 2.12* | (0.01,0.34) |
| **Mediation chains** |  |  |  |  |
| A1: Parents’ stress → child 1 wellbeing at t1 mediated by spousal relationship^a^ at t1 | -0.26 (0.15) | -0.22 | -1.76 | (-0.54,0.03) |
|  | -0.26 (0.14) | -0.22 | -1.85 | (-0.53,0.01) |
| A2: Parents’ stress → child 2 wellbeing at t1 mediated by spousal relationship^a^ at t1 | -0.25 (0.16) | -0.21 | -1.56 | (-0.57,0.06) |
|  | -0.25 (0.14) | -0.21 | -1.86 | (-0.52,0.01) |
| B1: Parents’ stress → child 1 wellbeing mediated at t1 by parent-child 1 relationship at t1 | 0.00 (0.07) | 0.00 | -0.02 | (-0.13,0.13) |
|  | 0.00 (0.06) | 0.00 | -0.02 | (-0.11,0.11) |
| B2: Parents’ stress → child 2 wellbeing at t1 mediated by parent-child 2 relationship at t1 | 0.05 (0.05) | 0.04 | 1.00 | (-0.05,0.15) |
|  | 0.05 (0.04) | 0.04 | 1.15 | (-0.04,0.14) |

MLR estimation: *n* = 96; Model fit measures: CFI = 0.911; RMSEA = 0.062; SRMR = 0.075. * *p* < .05 ** *p* < .01 *** *p* < .001.

MLM estimation: *n* = 96; Model fit measures: CFI = 0.915; RMSEA = 0.063; SRMR = 0.075. * *p* < .05 ** *p* < .01 *** *p* < .001**.**

^a^ Higher values reflect higher levels of spousal relationship problems.
