# Supplementary material for: COVID-19’s shadow on families: A structural equation model of parental stress, family relationships, and child wellbeing
Source: PLoS One. 2023 Oct 12;18(10):e0292292. doi: 10.1371/journal.pone.0292292 (PMC10569562; doi:10.1371/journal.pone.0292292)
Supplement: S4 Table — (DOCX) [file pone.0292292.s006.docx]

**S4 Table. Correlation between variables (full sample, n=783).**

|  | 1 | 2 | 3 | 4 | 5 | 6 | 7 | 8 | 9 | 10 | 11 | 12 | 13 | 14 |
| --- | --- | --- | --- | --- | --- | --- | --- | --- | --- | --- | --- | --- | --- | --- |
| 1. COVID-related economic pressure t1 | -- |  |  |  |  |  |  |  |  |  |  |  |  |  |
| 2. COVID-related health risks t1 | 0.33 | -- |  |  |  |  |  |  |  |  |  |  |  |  |
| 3. COVID-related job loss t1 | 0.26 | -0.03 | -- |  |  |  |  |  |  |  |  |  |  |  |
| 4. Child wellbeing t1 | -0.23 | -0.12 | -0.20 | -- |  |  |  |  |  |  |  |  |  |  |
| 5. Parents’ stress t1 | 0.20 | 0.18 | 0.09 | -0.28 | -- |  |  |  |  |  |  |  |  |  |
| 6. Spousal relationship^a^ t1 | 0.18 | 0.10 | 0.09 | -0.23 | 0.41 | -- |  |  |  |  |  |  |  |  |
| 7. Parent-child relationship t1 | -0.13 | -0.18 | 0.01 | 0.38 | -0.20 | -0.15 | -- |  |  |  |  |  |  |  |
| 8. COVID-related economic pressure t2 | 0.31 | 0.00 | 0.28 | -022 | 0.07 | 0.10 | -0.05 | -- |  |  |  |  |  |  |
| 9. COVID-related health risks t2 | 0.05 | 0.29 | 0.06 | -0.09 | 0.13 | 0.10 | -0.05 | 0.41 | -- |  |  |  |  |  |
| 10. COVID-related job loss t2 | 0.16 | -0.13 | 0.42 | -0.07 | 0.06 | 0.04 | 0.01 | 0.22 | -0.06 | -- |  |  |  |  |
| 11. Child 1 wellbeing t2 | -0.15 | -0.11 | -0.11 | 0.40 | -0.28 | -0.21 | 0.26 | -0.08 | 0.01 | -0.12 | -- |  |  |  |
| 12. Parents’ stress t2 | 0.10 | 0.11 | 0.04 | -0.12 | 0.37 | 0.28 | -0.18 | -0.05 | -0.15 | 0.08 | -0.42 | -- |  |  |
| 13. Spousal relationship^a^ t2 | 0.13 | 0.11 | 0.09 | -0.18 | 0.28 | 0.54 | -0.23 | 0.01 | 0.01 | 0.05 | -0.37 | 0.52 | -- |  |
| 14. Parent-child relationship t2 | -0.08 | -0.16 | -0.01 | 0.22 | -0.18 | -0.13 | 0.46 | -0.02 | -0.01 | 0.00 | 0.50 | -0.39 | -0.31 | -- |
| Mean | 51.56 | 40.87 | 0.22 | 3.62 | 2.62 | 2.45 | 1.44 | 44.99 | 42.48 | 0.19 | 3.39 | 2.49 | 2.41 | 0.82 |
| SD | 26.19 | 20.86 | 0.41 | 0.97 | 0.71 | 1.09 | 1.36 | 29.79 | 24.16 | 0.39 | 0.97 | 0.75 | 1.11 | 1.40 |

^a^ Higher values reflect higher levels of spousal relationship problems.
