# Supplementary material for: COVID-19’s shadow on families: A structural equation model of parental stress, family relationships, and child wellbeing
Source: PLoS One. 2023 Oct 12;18(10):e0292292. doi: 10.1371/journal.pone.0292292 (PMC10569562; doi:10.1371/journal.pone.0292292)
Supplement: S5 Table — (DOCX) [file pone.0292292.s007.docx]

**S5 Table. Results of the structural equation modeling testing assumptions of the FSM (first row: MLR estimation; second row: MLM estimation).**

| *Effect* | *β (SE)* | *std β* | *z-value* | *95% CI* |
| --- | --- | --- | --- | --- |
| Cross-sectional effects at t1 | | | | |
| Predictors |  |  |  |  |
| COVID-19 related economic pressure → Parents’ stress | 0.26 (0.06) | 0.21 | 4.63*** | (0.15,0.37) |
|  | 0.31 (0.03) | 0.31 | 10.38*** | (0.25,0.37) |
| COVID-19 related job loss → Parents' stress | 0.07 (0.05) | 0.05 | 1.21 | (-0.04,0.17) |
|  | 0.30 (0.03) | 0.31 | 10.27*** | (0.24,0.36) |
| COVID-19 related health risks → Parents' stress | 0.17 (0.05) | 0.14 | 3.43*** | (0.07,0.26) |
|  | 0.36 (0.03) | 0.38 | 11.45*** | (0.30,0.43) |
| Parents’ stress → Spousal relationship^a^ | 0.35 (0.05) | 0.28 | 6.87*** | (0.25,0.45) |
|  | 0.35 (0.05) | 0.28 | 7.54*** | (0.26,0.44) |
| Parents’ stress → Parent-child relationship | -0.57 (0.08) | -0.31 | -7.36*** | (-0.73,-0.42) |
|  | -0.57 (0.07) | -0.31 | -7.70*** | (-0.72,-0.43) |
| Spousal relationship^a^ → Child wellbeing | -0.12 (0.04) | -0.12 | -2.74** | (-0.21,-0.03) |
|  | -0.12 (0.04) | -0.12 | -2.84*** | (-0.21,-0.04) |
| Parent-child relationship → Child wellbeing | 0.15 (0.03) | 0.22 | 5.63*** | (0.10,0.20) |
|  | 0.15 (0.03) | 0.22 | 5.55*** | (0.10,0.20) |
| Parents’ stress → Child wellbeing | -0.22 (0.05) | -0.17 | -4.19*** | (-0.32,-0.11) |
|  | -0.22 (0.06) | -0.17 | -3.79*** | (-0.33,-0.10) |
| Covariates |  |  |  |  |
| Nationality: UAE → Parents’ stress | 0.09 (0.05) | 0.07 | 1.67 | (-0.02,0.19) |
|  | 0.09 (0.05) | 0.07 | 1.75 | (-0.01,0.19) |
| Nationality: UAE → Spousal relationship^a^ | 0.02 (0.07) | 0.01 | 0.36 | (-0.10,0.15) |
|  | 0.02 (0.06) | 0.01 | 0.37 | (-0.10,0.15) |
| Nationality: UAE → Parent-child relationship | 0.08 (0.09) | 0.03 | 0.94 | (-0.09,0.25) |
|  | 0.08 (0.08) | 0.03 | 0.95 | (-0.08,0.24) |
| Nationality: UAE → Child wellbeing | 0.49 (0.06) | 0.29 | 8.57*** | (0.37,0.60) |
|  | 0.49 (0.06) | 0.29 | 8.57*** | (0.37,0.60) |
| Education: BA degree and higher → Parents’ stress | 0.01 (0.05) | 0.01 | 0.14 | (-0.09,0.10) |
|  | 0.01 (0.05) | 0.01 | 0.14 | (-0.09,0.10) |
| Education: BA degree and higher → Spousal relationship^a^ | 0.10 (0.06) | 0.06 | 1.64 | (-0.02,0.22) |
|  | 0.10 (0.06) | 0.06 | 1.63 | (-0.02,0.22) |
| Education: BA degree and higher → Parent-child relationship | -0.33 (0.08) | -0.14 | -4.13*** | (-0.48,-0.17) |
|  | -0.33 (0.08) | -0.14 | -4.18*** | (-0.48,-0.17) |
| Education: BA degree and higher → Child wellbeing | 0.07 (0.06) | 0.04 | 1.18 | (-0.04,0.18) |
|  | 0.07 (0.06) | 0.04 | 1.18 | (-0.04,0.18) |
| Parent’s age (std) → Parents’ stress | -0.22 (0.13) | -0.06 | -1.68 | (-0.48,0.04) |
|  | -0.22 (0.13) | -0.06 | -1.66 | (-0.49,0.04) |
| Parent’s age (std) → Spousal relationship^a^ | 0.08 (0.17) | 0.02 | 0.49 | (-0.25,0.42) |
|  | 0.08 (0.17) | 0.02 | 0.49 | (-0.25,0.42) |
| Child age (std) → Parent-child relationship | -0.10 (0.07) | -0.05 | -1.42 | (-0.24,0.04) |
|  | -0.10 (0.07) | -0.05 | -1.38 | (-0.25,0.04) |
| Child age (std) → Child wellbeing | -0.10 (0.05) | -0.07 | -1.90 | (-0.20,0.00) |
|  | -0.10 (0.05) | -0.07 | -1.87 | (-0.20,0.00) |
| Male child → Parent-child relationship | 0.00 (0.08) | 0.00 | -0.01 | (-0.15,0.15) |
|  | 0.00 (0.08) | 0.00 | -0.01 | (-0.15,0.15) |
| Male child → Child wellbeing | -0.01(0.06) | -0.01 | -0.15 | (-0.12,0.10) |
|  | -0.01 (0.06) | -0.01 | -0.15 | (-0.12,0.10) |
| Child’s outdoor activity → Child wellbeing | 0.06 (0.03) | 0.08 | 2.39* | (0.01,0.11) |
|  | 0.06 (0.03) | 0.08 | 2.39* | (0.01,0.11) |
| Cross sectional effects at t2 | | | | |
| Predictors |  |  |  |  |
| COVID-19 related economic pressure → Parents’ stress | 0.00 (0.06) | 0.00 | 0.03 | (-0.12,0.13) |
|  | 0.00 (0.06) | 0.00 | 0.03 | (-0.12,0.12) |
| COVID-19 related job loss → Parents’ stress | 0.05 (0.07) | 0.03 | 0.68 | (-0.09,0.18) |
|  | 0.05 (0.07) | 0.03 | 0.71 | (-0.08,0.18) |
| COVID-19 related health risks → Parents’ stress | -0.37 (0.06) | -0.26 | -6.03*** | (-0.49,-0.25) |
|  | -0.37 (0.06) | -0.26 | -6.31*** | (-0.49,-0.26) |
| Parents’ stress → Spousal relationship^a^ | 0.56 (0.06) | 0.40 | 10.05*** | (0.45,0.67) |
|  | 0.56 (0.05) | 0.40 | 10.50*** | (0.46,0.67) |
| Parents’ stress → Parent-child relationship | 0.28 (0.08) | 0.16 | 3.37*** | (0.12,0.45) |
|  | 0.28 (0.08) | 0.16 | 3.79*** | (0.14,0.43) |
| Spousal relationship^a^ → Child wellbeing | -0.16 (0.05) | -0.18 | -3.26*** | (-0.26,-0.07) |
|  | -0.16 (0.05) | -0.18 | -3.44*** | (-0.26,-0.07) |
| Parent-child relationship → Child wellbeing | 0.02 (0.02) | 0.03 | 0.79 | (-0.03,0.07) |
|  | 0.02 (0.02) | 0.03 | 0.82 | (-0.03,0.07) |
| Parents’ stress → Child wellbeing | -0.38 (0.07) | -0.30 | -5.81*** | (-0.51,-0.25) |
|  | -0.38 (0.06) | -0.30 | -6.38*** | (-0.50,-0.26) |
| Covariates |  |  |  |  |
| Nationality: UAE → Parents’ stress | -0.07 (0.05) | -0.05 | -1.33 | (-0.18,0.03) |
|  | -0.07 (0.05) | -0,05 | -1.36 | (-0.18,0.03) |
| Nationality: UAE → Spousal relationship^a^ | 0.01 (0.07) | 0.00 | 0.12 | (-0.12,0.14) |
|  | 0.01 (0.07) | 0.00 | 0.12 | (-0.12,0.14) |
| Nationality: UAE → Parent-child relationship | -0.05 (0.10) | -0.02 | -0.56 | (-0.25,0.14) |
|  | -0.05 (0.10) | -0.02 | -0.57 | (-0.24,0.13) |
| Nationality: UAE → Child wellbeing | -0.12 (0.07) | -0.06 | -1.61 | (-0.26,0.03) |
|  | -0.12 (0.07) | -0.06 | -1.67 | (-0.25,0.02) |
| Education: BA degree and higher → Parents’ stress | 0.29 (0.05) | 0.20 | 5.89*** | (0.19,0.39) |
|  | 0.29 (0.05) | 0.20 | 5.79*** | (0.19,0.39) |
| Education: BA degree and higher → Spousal relationship^a^ | 0.17 (0.07) | 0.08 | 2.58* | (0.04,0.30) |
|  | 0.17 (0.06) | 0.08 | 2.62** | (0.04,0.29) |
| Education: BA degree and higher → Parent-child relationship | 0.18 (0.09) | 0.07 | 2.03* | (0.01,0.36) |
|  | 0.18 (0.09) | 0.07 | 1.95* | (0.00,0.36) |
| Education: BA degree and higher → Child wellbeing | 0.05 (0.06) | 0.03 | 0.79 | (-0.07,0.17) |
|  | 0.05 (0.06) | 0.03 | 0.77 | (-0.08,0.17) |
| Parent’s age (std) → Parents’ stress | 0.02 (0.15) | 0.00 | 0.13 | (-0.27,0.30) |
|  | 0.02 (0.14) | 0.00 | 0.13 | (-0.26,0.30) |
| Parent’s age (std) → Spousal relationship^a^ | 0.21 (0.19) | 0.03 | 1.10 | (-0.16,0.57) |
|  | 0.21 (0.18) | 0.03 | 1.15 | (-0.15,0.56) |
| Child age (std) → Parent-child relationship | -0.09 (0.08) | -0.04 | -1.08 | (-0.25,0.07) |
|  | -0.09 (0.09) | -0.04 | -1.04 | (-0.26,0.08) |
| Child age (std) → Child wellbeing | -0.12 (0.06) | -0.07 | -2.08** | (-0.23,-0.01) |
|  | -0.12 (0.06) | -0.07 | -2.09* | (-0.23,-0.01) |
| Male child → Parent-child relationship | -0.07 (0.09) | -0.03 | -0.78 | (-0.24,0.10) |
|  | -0.07 (0.09) | -0.03 | -0.77 | (-0.24,0.11) |
| Male child → Child wellbeing | 0.02 (0.06) | 0.01 | 0.38 | (-0.09,0.14) |
|  | 0.02 (0.06) | 0.01 | 0.37 | (-0.09,0.14) |
| Child’s outdoor activity → Child wellbeing | 0.06 (0.03) | 0.07 | 2.07* | (0.11, 0.06) |
|  | 0.06 (0.03) | 0.07 | 2.04* | (0.00,0.11) |
| Lagged effects | | | | |
| Parents’ stress at t1 → Parents’ stress at t2 | 0.51 (0.06) | 0.42 | 8.77*** | (0.39,0.62) |
|  | 0.51 (0.05) | 0.42 | 9.54*** | (0.40,0.61) |
| Parents’ stress at t1 → Parent-child relationship at t2 | -0.18 (0.10) | -0.09 | -1.79 | (-0.38,0.02) |
|  | -0.18 (0.1) | -0.09 | -1.88 | (-0.38,0.01) |
| Parents’ stress at t1 → Spousal relationship^a^ at t2 | 0.35 (0.05) | 0.21 | 6.87*** | (0.25,0.45) |
|  | 0.35 (0.05) | 0.21 | 7.54*** | (0.26,0.44) |
| Parents’ stress at t1 → Child wellbeing at t2 | -0.08 (0.08) | -0.05 | -1.05 | (-0.23,0.07) |
|  | -0.08 (0.07) | -0.05 | -1.12 | (-0.22,0.06) |
| Parent-child relationship at t1 → Parent-child relationship at t2 | 0.27 (0.04) | 0.24 | 6.5*** | (0.19,0.35) |
|  | 0.27 (0.04) | 0.24 | 6.47*** | (0.19,0.35) |
| Parent-child relationship at t1 → Child wellbeing t2 | -0.02 (0.03) | -0.03 | -0.65 | (-0.08,0.04) |
|  | -0.02 (0.03) | -0.03 | -0.69 | (-0.08,0.04) |
| Spousal relationship^a^ at t1 → Spousal relationshipa at t2 | 0.46 (0.06) | 0.34 | 7.92*** | (0.34,0.57) |
|  | 0.46 (0.05) | 0.34 | 8.54*** | (0.35,0.56) |
| Spousal relationship^a^ at t1 → Child wellbeing at t2 | 0.08 (0.05) | 0.07 | 1.56 | (-0.02,0.19) |
|  | 0.08 (0.05) | 0.07 | 1.57 | (-0.02,0.19) |
| Child wellbeing at t1 → Child wellbeing at t2 | 0.47 (0.05) | 0.39 | 8.78*** | (0.37,0.58) |
|  | 0.47 (0.05) | 0.39 | 9.01*** | (0.37,0.58) |
| Mediation chains | | | | |
| t1-A1: Parents’ stress at t1 → child wellbeing at t1 mediated by spousal relationship^a^ at t1 | -0.04 (0.02) | -0.03 | -2.60** | (-0.08,-0.01) |
|  | -0.04 (0.02) | -0.03 | -2.71** | (-0.07,-0.01) |
| t2-A2: Parents’ stress at t2 → child wellbeing at t2 mediated by spousal relationship^a^ at t2 | -0.09 (0.03) | -0.07 | -3.24*** | (-0.15,-0.04) |
|  | -0.09 (0.03) | -0.07 | -3.34*** | (-0.15,-0.04) |
| t1-A3: Parents’ stress at t1 → child wellbeing at t1 mediated by parent-child relationship at t1 | -0.08 (0.02) | -0.07 | -4.85*** | (-0.12,-0.05) |
|  | -0.08 (0.02) | -0.07 | -4.58*** | (-0.12,-0.05) |
| t2-A4: Parents’ stress at t2 → child wellbeing at t1 mediated by parent-child relationship at t2 | 0.01 (0.01) | 0.00 | 0.77 | (-0.01,0.02) |
|  | 0.01 (0.01) | 0.00 | 0.79 | (-0.01,0.02) |
| t1-B1: Parents’ stress at t1 → child wellbeing at t2 mediated by spousal relationship^a^ at t1 | 0.03 (0.02) | 0.02 | 1.53 | (-0.01,0.07) |
|  | 0.03 (0.02) | 0.02 | 1.54 | (-0.01,0.07) |
| t2-B2: Parents’ stress at t1 → child wellbeing at t2 mediated by spousal relationship^a^ at t2 | -0.06 (0.02) | -0.05 | -2.95*** | (-0.10,-0.02) |
|  | -0.06 (0.02) | -0.05 | -3.15*** | (-0.09,-0.02) |
| t1-B3: Parents’ stress at t1 → child wellbeing at t2 mediated by parent child relationship at t1 | 0.01 (0.02) | 0.01 | 0.65 | (-0.02,0.05) |
|  | 0.01 (0.02) | 0.01 | 0.69 | (-0.02,0.05) |
| t2-B4: Parents’ stress at t1 → child wellbeing at t2 mediated by parent child relationship at t2 | 0.00 (0.00) | 0.00 | -0.73 | (-0.01,0.01) |
|  | 0.00 (0.00) | 0.00 | -0.75 | (-0.01,0.01) |

MLR estimation: *n* = 783; Model fit measures: CFI = 0.906; RMSEA = 0.048; SRMR = 0.062. * *p* < .05 ** *p* < .01 *** *p* < .001.

MLM estimation: *n* = 783; Model fit measures: CFI = 0.911; RMSEA = 0.047; SRMR = 0.060. * *p* < .05 ** *p* < .01 *** *p* < .001**.**

^a^ Higher values reflect higher levels of spousal relationship problems.
